# On the role of subsecond dopamine release in conditioned avoidance

**DOI:** 10.3389/fnins.2013.00096

**Published:** 2013-06-07

**Authors:** Erik B. Oleson, Joseph F. Cheer

**Affiliations:** Department of Anatomy and Neurobiology, School of Medicine, University of MarylandBaltimore, MD, USA

**Keywords:** dopamine, voltammetry, conditioned avoidance, nucleus accumbens, fear conditioning

## Abstract

Using shock avoidance procedures to study conditioned behavioral responses has a rich history within the field of experimental psychology. Such experiments led to the formulation of the general concept of negative reinforcement and specific theories attempting to explain escape and avoidance behavior, or why animals choose to either terminate or prevent the presentation of an aversive event. For example, the two-factor theory of avoidance holds that cues preceding an aversive event begin to evoke conditioned fear responses, and these conditioned fear responses reinforce the instrumental avoidance response. Current neuroscientific advances are providing new perspectives into this historical literature. Due to its well-established role in reinforcement processes and behavioral control, the mesolimbic dopamine system presented itself as a logical starting point in the search for neural correlates of avoidance and escape behavior. We recently demonstrated that phasic dopamine release events are inhibited by stimuli associated with aversive events but increased by stimuli preceding the successful avoidance of the aversive event. The latter observation is inconsistent with the second component of the two-factor theory of avoidance and; therefore, led us propose a new theoretical explanation of conditioned avoidance: (1) fear is initially conditioned to the warning signal and dopamine computes this fear association as a decrease in release, (2) the warning signal, now capable of producing a negative emotional state, suppresses dopamine release and behavior, (3) over repeated trials the warning signal becomes associated with safety rather than fear; dopaminergic neurons already compute safety as an increase in release and begin to encode the warning signal as the earliest predictor of safety (4) the warning signal now promotes conditioned avoidance *via* dopaminergic modulation of the brain's incentive-motivational circuitry.

## Introduction to conditioned avoidance

**Figure 1:**
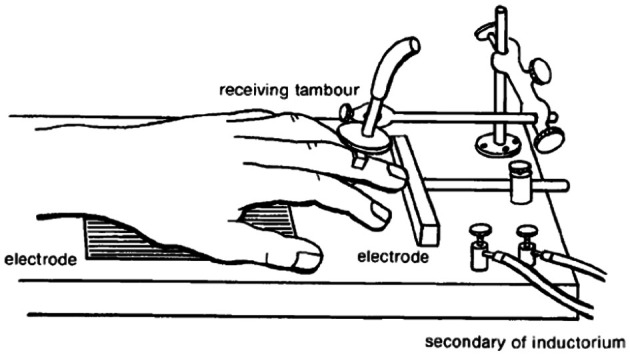
**Illustration of the first conditioned avoidance (initially described as a conditioned reflex) experiment conducted in America by John B Watson**. Electrodes were placed under the hand and finger of a human subject. An auditory stimulus was presented prior to the delivery of electrical shock. A recording device allowed for the detection of finger movements evoked by the shock and the preceding auditory stimulus. Within a few trials, finger withdrawal began to occur to the auditory stimulus. The conditioned finger withdrawal broke the circuit between the two electrodes, which was necessary for the delivery of electric shock. Originally published in Watson (1916).

Conditioned avoidance is an acquired behavioral response that results in the prevention of an aversive event. Conditioned avoidance was first described by one of Ivan Pavlov's chief scientific rivals, Bechterev (1913) before being introduced to American psychology by Watson (1916). Ironically, Watson adopted Bekhterev's experimental approach of investigating “associated” motoric avoidance responses in an attempt to validate Pavlov's work on classical conditioning (Bolles, 1972). While it is well known that Pavlov clearly demonstrated that dogs exhibit a strong salivary reflex to stimuli previously associated with food (Pavlov, 2003), Watson found odor-evoked conditional reflexes of the human parotid gland to be elusive (Lashley, 1916; Watson, 1916). Thus, in an attempt to observe a conditioned reflex in human subjects, Watson turned to Bekhterev's experimental design (Figure 1), in which: electrodes capable of delivery faradaic stimulation are placed under the palm and finger of a human subject, the hand is exposed to a mild electrical shock that is preceded by a bell (2 s prior to shock), finger movement eliminates electric shock by breaking the circuit between the two electrodes, motoric finger responses are measured by a lever that supports a writing lever (Bechterev, 1913; Watson, 1916). Under these conditions, finger withdrawal initially occurred in response to the electric shock, but within a few trials finger withdrawal began to occur to the bell—thereby leading to the complete avoidance of electric shock (Watson, 1916). This conditioned behavioral response to a shock-predictive cue proved to be highly replicable across subjects, ages and species (Watson, 1916). Although Watson interpreted the aforementioned response as a conditioned reflex, today we recognize this behavioral action as a conditioned avoidance response that is energized by the incentive-motivational circuitry of the brain. One of the major theories involved in integrating motivational theory with conditioned avoidance is the two-process theory of avoidance (Miller, 1948; Mowrer and Aiken, 1954). In general, this theory holds that conditioned fear responses resulting from Pavlovian learning motivate avoidance behavior through fear reduction. The first factor of this theory describes the Pavlovian associations that are established between the aversive stimulus (shock) and the preceding cue (the bell in Watson's experiment). The second factor of this theory states that the fear evoked by the preceding cue functions to reinforce the avoidance response. Over the course of the century, investigators developed various methodological adaptations to study conditioned avoidance using experimental animals, most prominently shuttle boxes (Warner, 1932) and operant chambers (Skinner, 1938). It is not the intention of this review to focus on theoretical intricacies of avoidance learning. Instead, we would like to refer the reader to recent reviews focusing on the learning mechanisms that might contribute to the development of avoidance behavior (Depue and Collins, 1999; Moutoussis et al., 2008; Maia, 2010). The present review will focus primarily on the role of the mesolimbic dopamine system during behavior maintained in a signaled operant avoidance procedure. In particular, we will describe how subsecond dopamine release relates to discrete cues during conditioned avoidance and escape responses. Here, it is critical to understand the distinction between avoidance and escape responses. Specifically, an escape response is defined as an action resulting in the cessation of an ongoing aversive stimulus; whereas, an avoidance response is defined as an action preventing the presentation of the aversive stimulus. Two discrete cues will be discussed. A warning signal (a cue light in our case) predicts the potential occurrence of an aversive event; a safety signal (a tone in our case) indicates that the aversive event was successfully avoided or terminated.

## Dopamine, incentive motivation and conditioned avoidance

When experimental psychologists began considering the phenomenon of conditioned avoidance in the middle of the twentieth century, they were relatively unsatisfied with Watson's interpretation that the avoidance response is simply a conditioned reflex resulting from classical conditioning (Bolles, 1972). Alternative explanations began to emerge, many of which described conditioned avoidance as a reinforcement process influenced by the experimental subject's motivation to avoid or terminate the aversive stimulus (e.g., Miller, 1948; Mowrer and Aiken, 1954). The purely psychological view that incentive-motivation (defined as the energizing effects of an encounter with an otherwise neutral stimulus that has acquired motivational importance through prior association, Wise, 2004) might influence the maintenance of conditioned avoidance is supported by modern neuroscientific research.

Before we discuss a role for subsecond dopamine release in conditioned avoidance, it is important to first briefly overview the neural circuitry involved in centrally representing incentive salience. One of the most studied components of the motivational circuitry of the brain is the nucleus accumbens. This brain region has been referred to as a limbic-motor (Mogenson et al., 1980) and Pavlovian-instrumental (Cardinal et al., 2002) interface—both of which appropriately represent the importance of the nucleus accumbens during an avoidance task in which a subject's behavior is effected by their motivational state and conditioned predictors of aversive stimuli. Of note, the nucleus accumbens integrates input from amygdalar and prefrontal cortical regions that carry information regarding the motivational value of stimuli maintaining reinforcement processing before energizing ongoing behavior (Cardinal et al., 2002). The mesolimbic dopamine pathway is theorized to modulate the integration of these motivational circuits by stamping-in stimulus-reinforcement associations, thereby strengthening the incentive value ascribed to previously neutral stimuli (e.g., warning signal) and motivating the conditioned behavioral response (Wise, 2004), or in this case conditioned avoidance.

The mesolimbic dopamine system is a neural pathway that originates from A10 dopamine neurons in the ventral tegmental area of the midbrain and projects to the brain's motivational circuitry, most prominently the nucleus accumbens, amygdala and prefrontal cortices (Swanson, 1982; Spanagel and Weiss, 1999). During ongoing behavior, two distinct patterns of dopamine release occur. Midbrain dopamine neurons typically fire at low frequencies of 1–5 Hz, which is thought to produce a tone on high affinity dopamine D2 receptors in the nucleus accumbens (Grace, 1991; Dreyer et al., 2010). Experimentally, one can detect tonic dopamine levels using techniques like *in vivo* microdialysis, which allow for neurochemical detection on a timescale of minutes. In contrast, when animals are presented with motivationally salient stimuli, A10 dopamine neurons fire in high frequency bursts (≥20 Hz). These high frequency bursts of dopaminergic neural activity produce transient increases in dopamine concentration in terminal fields (e.g., nucleus accumbens). Dopamine concentration transients are detectable at the neurochemical level within terminal fields of the mesolimbic dopamine system using fast-scan cyclic voltammetry, an electrochemical technique that allows for the detection of dopamine on the millisecond timescale. Importantly, only neurochemical techniques like fast-scan cyclic voltammetry provide the temporal resolution necessary to measure dopamine release events evoked by a warning signal in a standard conditioned avoidance procedure.

Pharmacological, lesion, genetic and microdialysis studies have been conducted over the last few decades to demonstrate a general role for dopamine in conditioned avoidance. Animals fail to acquire avoidance following 6-hydroxydopamine lesions of midbrain dopamine neurons, a deficit that is reversed by the restoration of dopamine levels using L-dopa treatment (Cooper et al., 1973; Zis et al., 1974). Intriguingly, only deficits in avoidance responses are observed, as opposed to responses motivated by the termination of ongoing shock (i.e., escape responses) (Fibiger et al., 1975). Similar observations are reported during the maintenance of conditioned avoidance. Lesions of dopamine terminals in the striatum in general (Amalric and Koob, 1987) and ventral striatum (i.e., nucleus accumbens) in particular (McCullough et al., 1993) are sufficient to impair conditioned avoidance. Systemic administration of dopamine receptor antagonists reliably disrupts avoidance responding without significantly impairing escape behavior (Arnt, 1982). Likewise, locally infusing a dopamine receptor antagonist into the nucleus accumbens alone is sufficient to impair the maintenance of conditioned avoidance (Wadenberg et al., 1990). Using recently developed genetic technology (Darvas et al., 2011) restored dopamine in specific brain regions that were otherwise dopamine-deficient. They found that while the entire striatum and amygdala are necessary for the acquisition of conditioned avoidance, only the striatum is required for the maintenance of conditioned avoidance (Darvas et al., 2011). These findings are in agreement with previous work demonstrating that the amygdala, while important for aversively motivated learning (Ledoux and Muller, 1997; LeDoux, 2003), plays a more specific role in the acquisition rather than the maintenance of instrumental avoidance behavior (Poremba and Gabriel, 1999). In addition to the amygdala and nucleus accumbens, it is important to note that the Gabriel lab has discovered that cingular-thalamic circuitry is also necessary for avoidance learning (Gabriel, 1993). For example, lesions of the anterior cingulate cortex or the limbic thalamus impair acquisition of conditioned avoidance (Gabriel et al., 1989, 1991). Microdialysis studies have demonstrated that dopamine levels are generally increased in the prefrontal cortex and striatum during the acquisition (Dombrowski et al., 2012) and maintenance (McCullough et al., 1993; Feenstra et al., 2001) of conditioned avoidance. Together these studies demonstrated that dopamine plays a general role in the maintenance of conditioned avoidance.

Recently, Kapur (2003), Kapur et al. (2005) generated an incentive-motivation based theory that offers a specific role for dopamine in conditioned avoidance as they attempted to explain why antipsychotics are efficacious in modulating conditioned avoidance. Their theory is based on the observation that all effective antipsychotics antagonize dopamine D2 receptors and disrupt conditioned avoidance. In fact, conditioned avoidance is a classic animal model used to screen for the efficacy of antipsychotic drugs and their dopamine antagonizing properties (Kapur et al., 2005; Smith et al., 2005). This observation led these investigators to speculate that the development of a hyperdopaminergic state in schizophrenia leads to an aberrant assignment of incentive salience to environmental stimuli, thereby promoting psychosis (Kapur, 2003), and the effectiveness of antipsychotics to disrupt conditioned avoidance is due to their ability to block subsecond dopaminergic encoding of the warning signal after it has acquired incentive value (Kapur et al., 2005; Smith et al., 2005). If this theory is correct, discrete dopamine release events time-locked to the warning signal should be detected during the maintenance of conditioned avoidance.

## Subsecond dopamine release during warning signal presentation

**Figure 2:**
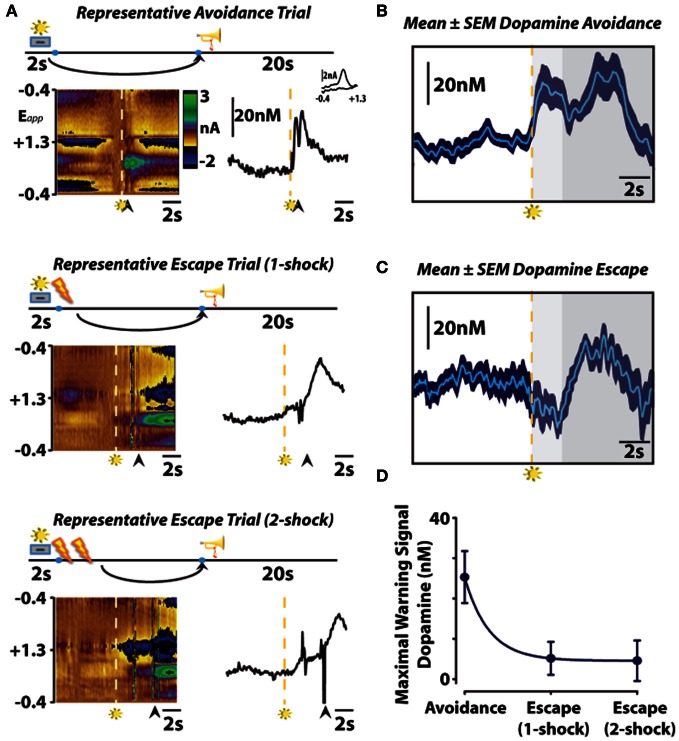
**The role of subsecond dopamine release during conditioned avoidance. (A)** Changes in subsecond dopamine release observed in different response types observed in a single session. Representative color plots (left) and dopamine concentration traces (right) show avoidance (top), one-footshock escape (middle), and two-footshock escape (bottom) responses. Left, the *y*-axis represents the scan potential (E*pp*, V) applied to the electrode, the *x*-axis represents time, and the *z*-axis represents current. Inspection of the color plot allows for the identification of dopamine over time. Dopamine can be identified in the color plot by assessing for changes in current at the oxidation (+0.6V) and reduction (−0.2V) potentials for dopamine. Right, representative dopamine concentration traces plotted as a function of time with the inset showing the cyclic voltammograms for dopamine. Arrows indicate lever responses, lightning bolts indicate footshocks, trumpets indicate safety periods, levers + lights indicate warning signals. **(B,C)** Mean ± SEM dopamine concentration traces from all avoidance and escape responses. Maximal warning signal duration is representative by the light gray fill, subsequent safety periods are represented by the dark gray fill. **(D)** Maximal dopamine concentration evoked by warning signal presentation predicts conditioned avoidance. Originally published in Oleson et al. (2012).

To investigate whether subsecond dopamine release is altered by the presentation of a warning signal, we used fast-scan cyclic voltammetry to assess subsecond dopaminergic release events in the nucleus accumbens core during behavior maintained in an operant signaled shock avoidance procedure (Figure 2). In this task, a stimulus light was presented as a warning signal for 2 s prior to the delivery of recurring foot shocks. During this 2 s warning signal, a response lever was extended into an operant chamber which, if depressed, resulted in the immediate retraction of the lever and a 20 s safety period signaled by a tone (i.e., safety signal). Animals could initiate an avoidance response by pressing the lever during the 2 s warning signal, entirely preventing shock. Alternatively, once shocks commenced, animals could initiate an escape response by pressing the lever during this punishment period, terminating shock. This experimental design allowed us to assess dopamine signaling during warning signal presentation, safety periods and during two distinct behavioral responses—avoidance and escape. It is important to note that, regardless of the methodology used (i.e., operant or shuttle box), avoidance and escape responses are distinct. This distinction was originally noted in one of the first conditioned avoidance experiments using a shuttle box with a hurdle that separated a shock-free side from a shock side (Bolles, 1972). In this early study, Warner reported that animals would scramble under the hurdle during escape responses, but jump over the hurdle during avoidance responses (Warner, 1932). He further went on to study the unique behavioral responses produced independently by either the shock or the warning signal and found that the shock produced scampering reactions whereas the warning signal produced more calculated, coordinated reactions (Warner, 1932). In the operant signaled shock avoidance task used in our study, we also observed distinct escape and avoidance reactions. Early in training, during which only operant escape responses occur, we observed several unique behavior reactions to the shock: jumping up the wall, attacking the lever and freezing. Interestingly, an unintentional (i.e., not experimenter intended outcome) avoidance response sometimes emerged early in training as well. In certain instances animals attempted to avoid shock by grounding themselves. As in Watson's early finger avoidance study (1916), electrical continuity is only maintained if the rat is in contact between two electrodes or, in our case, two electrified bars comprising the grid floor of the operant chamber. Occasionally, animals balanced their hind paws on a single bar while propping their front paws on a side of the operant chamber, thereby breaking the continuity of the electrical circuit and avoiding footshock. As the contingencies of reinforcement were learned, however, these unintended behaviors begin to dissipate until consistently maintained avoidance and escape behaviors emerged. In our first study on this subject (Oleson et al., 2012), we only recorded dopamine from animals in our operant avoidance task after they began avoiding footshock in ~50% of trials. At this point in training, we visually observed one of two distinct behavioral reactions in response to warning signal presentation. When the animal successfully avoided footshock, an uninhibited motor sequence directed at the lever was observed upon presentation of the warning signal. When the animal escaped footshock, a hesitation—presumably a fear-induced freezing response—was observed upon presentation of the warning signal. While it is well established that amygdalar modulation of prefrontal cortical activity is critically important in the expression of conditioned fear (Davis, 1992; Morgan and LeDoux, 1995; Garcia et al., 1999), dopaminergic modulation of striatal input may be involved in the expression of the freezing response. The canonical view of the basal ganglia holds that the striatum outputs two parallel projections, the direct and indirect pathways, which either excite or inhibit behavioral activity, respectively. According to this canonical view, dopamine release events are theorized to promote behavioral activation by increasing activity along the direct pathway by acting on G_s_ coupled dopamine D1 receptors, whereas decreases in dopamine release may inhibit behavioral activation by increasing activity along the indirect pathway by acting on G_i/o_ coupled dopamine D2 receptors (DeLong and Wichmann, 2007). A recent optogenetic study supported this conceptualization by demonstrating that selective activation of striatal dopamine D1 receptor expressing neurons of the direct pathway promotes behavioral activation, while selective activation of striatal dopamine D2 receptor expressing neurons of the indirect pathway promotes freezing behavior (Kravitz et al., 2010). Thus, it is possible that dopamine may contribute to the expression of a freezing response, although additional optogentic studies should be conducted to directly assess for this possibility within the context of conditioned fear. It is also important to note that, rather than solely causing avoidance or freezing responses by activating dopamine D1 or D2 receptors, dopamine concentration changes within the striatum are thought to modulate converging amygdalar, hippocampal and prefrontal input (Floresco et al., 2001; Brady and O'Donnell, 2004) to control behavioral activation.

**Figure 3:**
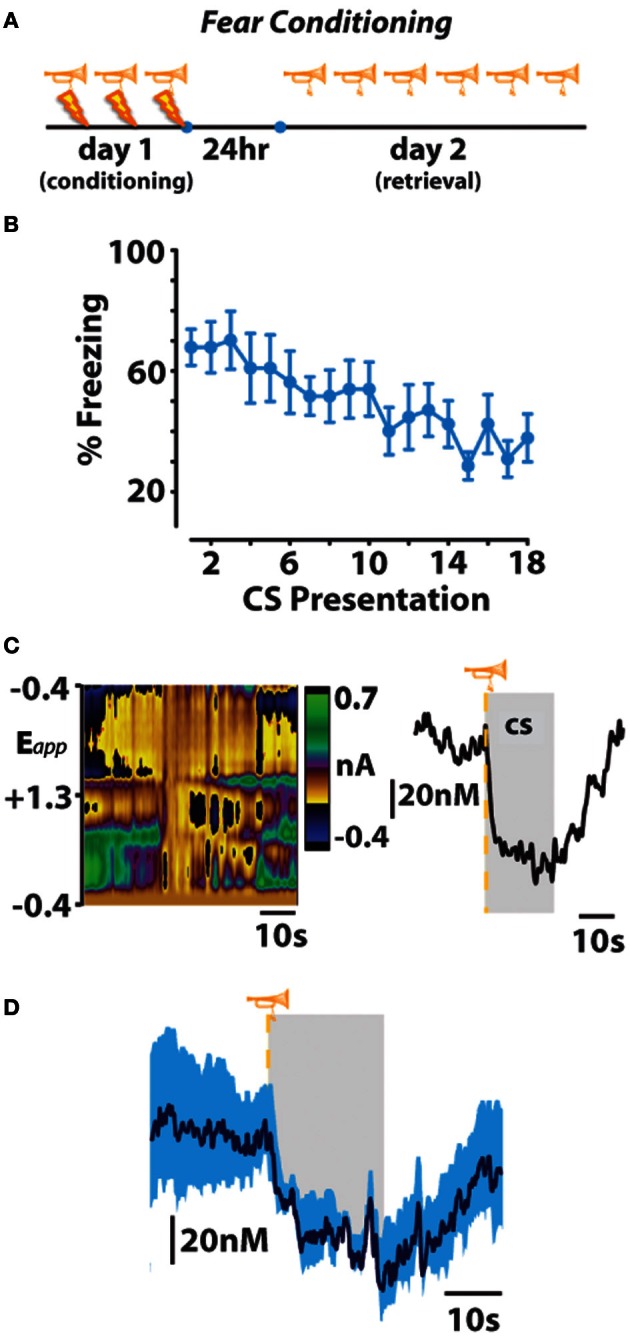
**Fear-conditioned stimuli freeze behavior and subsecond dopamine release events. (A,B)** An otherwise neutral stimulus (trumpet) previously conditioned to inescapable footshock (lightning bolt) produces freezing behavior that extinguishes across repeated trials of conditioned stimulus (CS) presentation on fear-memory retrieval day. **(C)** Representative color plot (left) and corresponding dopamine concentration trace (right) show a CS-induced decrease in dopamine release. Gray represents CS duration. **(D)** Mean ± SEM dopamine concentration trace during presentations of the fear-conditioned CS. Originally published in Oleson et al. (2012).

As animals displayed either directed avoidance or inhibited freezing responses to warning signal presentation, it might be expected, therefore that distinct dopaminergic responses accompany these divergent behavioral reactions. In accordance with our behavioral observation, dichotomous dopaminergic responses occurred at the warning signal during avoidance and escape behavior. When animals successfully avoided footshock, dopamine release increased during warning signal presentation as would be predicted if dopamine was motivating the avoidance response. Importantly, the warning signal evoked increase in dopamine concentration reliably predicted when an animal would successfully avoid foot shock. Trial-by-trial analysis revealed that the maximal dopamine concentration time-locked to warning signal presentation sharply decreased during trials in which animals failed to avoid and was significantly lower during escape responses irrespective of the number of footshocks received. Averaging dopamine concentrations during escape trials revealed that dopamine levels not only failed to increase during presentation of the warning signal presentation, dopamine release events actually ceased at warning signal onset when the animals failed to avoid. This latter finding is somewhat reminiscent of the previously described classical psychological theory called the two-process theory of avoidance (Mowrer, 1951). The first factor of this theory posits that fear becomes conditioned to the warning signal; the second factor suggests that the conditioned fear that is evoked by the warning signal is what reinforces the instrumental avoidance response *via* fear reduction. To further test whether our dopamine data align with the first-factor of this theory, we measured whether dopamine release in the nucleus accumbens core is also suppressed during classical fear associations by employing a standard fear-conditioning model. In this fear-conditioned model, animals were conditioned to an auditory stimulus predicting inescapable footshock before we measured dopamine release 24 h later during repeated presentations of the cue alone (Figure 3). As was observed at the warning-signal during escape responses, the fear-associated auditory stimulus produced a decrease in dopamine concentration transients (Oleson et al., 2012), a phenomenon that appears to be exclusive to the core, as opposed to the shell, subregion of the nucleus accumbens (Badrinarayan et al., 2012). This finding supports the first factor of the two-process theory of avoidance that the warning signal can evoke conditioned fear responses, and reveals that dopamine neurons compute this conditioned fear response as a decrease in the frequency of dopamine release events. These data fail to align with the second factor of two-process theory, however, as dopamine release accompanies the presentation of the warning signal when animals successfully avoid foot shock. Rather, fear may become irrelevant during conditioned avoidance in a well-trained animal. The warning signal no longer evokes fear, and fear reduction is no longer the primary motivator of behavior. Instead of evoking a fear response, the warning signal becomes associated exclusively with a positive outcome—avoidance. At this point the warning signal motivates behavior similarly to a reward-predictive cue, by stimulating the incentive-motivational circuitry of the brain.

The observation that dopamine begins to increase to the warning signal during avoidance trials suggests that the fear response originally elicited by the warning signal can dissipate over time, as the prediction of a positive outcome (i.e., successful avoidance) becomes more prominent. These findings support other recent work demonstrating that the representation of a conditioned cue can switch between appetitive and aversive stimuli over repeated pairings (Nasser and McNally, 2012) and was predicted by early experimental psychologists. In fact, it has long been reported that animals become less fearful during conditioned avoidance. In one of Richard Solomon's early experiments studying the extinction of the avoidance response, he noted that the animals “learn to relax” in the presence of the warning signal (Solomon et al., 1953). The possibility that the fear response evoked by the warning signal begins to dissipate over time was objectively tested in a subsequent study (Kamin et al., 1963), in which: rats were trained to respond for food in an operant chamber, then trained to avoid shock by responding to an auditory warning signal in a shuttle-box for either 1, 3, 9, or 27 trials, then retested in the operant chamber while periodic presentations of the warning signal occurred during food maintained responding. It was found that the warning signal was less effective at suppressing food maintained responding after 27 trials of conditioned avoidance in comparison to animals with less extensive behavioral histories (Kamin et al., 1963). Importantly, in each of these examples, the fear response evoked by the conditioned stimulus begins to dissipate while the avoidance response remains strong—so strong it is incredibly difficult to extinguish (Solomon et al., 1953). Thus, fear is unlikely to motivate effective avoidance responses in the well-trained rat. Instead, we propose that the strength of the avoidance response is bolstered by increases in dopamine release evoked by the warning signal through higher order reinforcement processes, and these warning signal evoked dopamine release events are capable of motivating avoidance behavior by modulating the incentive-motivational circuitry of the brain. It is also possible that these warning signal evoked dopamine release events might contribute to stimulus-response, or habit, learning. Habit learning reflects the formation of higher order stimulus-response associations (e.g., warning signal-avoidance) that are capable of reinforcing behavioral action but do not become encoded as a goal themselves; thus, rendering the behavior resistant to extinction despite primary reinforcer devaluation (Everitt and Robbins, 2005). Under these circumstances, dopaminergic encoding of the warning signal likely remains critical for the maintenance of conditioned avoidance, although a hierarchical shift of warning-signal evoked dopamine release toward brain regions more implicated in habitual behavior (e.g., dorsal striatum) may contribute (Willuhn et al., 2012). However, a transition to habit formation in this particular behavior may be critically linked to the animal's training history and may also be influenced by individual differences. For example, an animal whose responding is completely dominated by avoidance behavior may always respond to the warning signal even if the shock is removed. On the other hand, an animal that primarily shows escape behavior will extinguish responding when the shock is discontinued because the unconditioned stimulus is the primary driver of the action.

## Subsecond dopamine release during safety signal presentation

As occurs following the presentation of rewarding stimuli (Schultz et al., 1997), we observed an increase in dopamine release during the safety signal that was indistinguishable between avoidance and escape responses (Figure 2). Thus, the elimination of aversive stimuli is processed by dopamine neurons similarly to the receipt of reward, regardless of the representation of the preceding warning signal or whether or not foot shock actually occurred. These data are in agreement with recently published work showing that the relief of pain increases dopamine release in the nucleus accumbens (Navratilova et al., 2012), and further support the notion that avoidance or removal of negative stimuli produces negative reinforcement via mesolimbic dopamine release. This finding supports the theory that the safety signal acquires positive reinforcement value that is capable of promoting avoidance behavior by functioning as a positive conditioned reinforcer (Dinsmoor, 1954, 2001). Several previous studies directly assessed the positive reinforcing effectiveness of the safety signal. Early reports demonstrated that a tone, previously associated with a safety period, is capable of increasing rates of responding to a frequency required to produce the tone alone (Weisman and Litner, 1969). Dinsmoor and colleagues extended upon this finding by demonstrating that presentation of a conditioned safety signal increased rates of responding in a shock avoidance task in which the reinforcing operandum remained available between aversive events (Dinsmoor and Sears, 1973). Rescorla (1969) further proved the reinforcing strength the safety signal holds over avoidance behavior by showing that animals choose a shock-terminating operandum that produces a safety signal over one that simply stops shock. Together, these studies suggest that the safety signal acquires positive reinforcing value capable of promoting avoidance, and dopamine release encodes safety as an increase in release. However, it should also be noted that the warning signal and its dopaminergic correlate is a stronger determinant of the behavioral action than the safety signal and its dopaminergic correlate. That is, only the warning signal evoked dopamine concentration predicts an animal's behavioral response, as dopamine increased during the safety signal regardless of whether safety was reached by escape or avoidance of footshock.

## Tonic vs. phasic dopamine

All neurochemical data introduced within the subsequent two sections describe subsecond dopamine release events resulting from the phasic activation of A10 dopamine neurons. It is important to note that these phasic dopamine data are distinct from previous accounts of tonic dopamine release obtained using microdialysis. For example, microdialysis studies report that tonic brain dopamine levels are generally increased during both conditioned avoidance (McCullough et al., 1993; Feenstra et al., 2001) and fear conditioning (Young et al., 1993; Wilkinson et al., 1998). As previously suggested (McGinty et al., 2011; Oleson et al., 2012), we believe these seemingly contradictory results can be explained by the possibility that aversive stimuli selectively suppress phasic dopamine release while concurrently enhancing tonic dopamine release. In this sense, tonic patterns of dopamine release may serve as an opponent-process (Solomon and Corbit, 1974) to phasic dopamine release evoked by aversive stimuli. It has also been suggested that phasic and tonic dopaminergic encoding of aversive stimuli might vary between subregions of the nucleus accumbens (Badrinarayan et al., 2012). Advances in microdialysis technology offering greater temporal and spatial resolution (Perry et al., 2009) will allow for the clarification of whether these relationships between phasic and tonic dopamine release exist.

## Synthesizing our neurochemical observations with the historical psychological literature led us to formulate the following 4-factor dopaminergic theory of signaled operant avoidance

1. As in the original two-process theory of avoidance, fear is initially conditioned to the warning signal and dopamine computes this fear association as a decrease in release.
2. The conditioned fear evoked by the warning signal elicits a freezing response, which actually inhibits operant avoidance.
3. Over repeated trials the warning signal becomes associated with safety rather than fear. Dopaminergic neurons already compute safety as an increase in release. Similarly to the temporal difference model of reinforcement learning (Schultz et al., 1997), dopamine release begins to encode the warning signal as the earliest predictor of safety through a positive prediction error, as the animal's expectation of a negative outcome (being shocked) is violated when avoidance takes place.
4. The warning signal now promotes conditioned avoidance *via* dopaminergic modulation of brain's incentive-motivational circuitry.

This new model, inspired by recent neurochemical findings, is based upon our conceptualization of the associative structure of the avoidance memory. Specifically, we speculate that early in training the safety signal is associated with the alleviation or avoidance of shock and the warning signal is associated with fear (as in the two-process theory of avoidance); later in training, the safety signal remains associated with the alleviation/avoidance of shock while the warning signal becomes associated with the successful avoidance of foot shock through a reinforcement learning mechanism. The role that temporal difference reinforcement learning may play in transition of cue-evoked dopamine from the safety signal to the warning signal during conditioned avoidance has been previously discussed in detail (Hollon et al., 2013). Briefly, temporal difference reinforcement learning is driven by the error between temporally successive predictions (Sutton, 1988) and midbrain dopamine neurons acquire reward-predicting responses to conditioned cues (Schultz et al., 1997). As detailed by Hollon et al. (2013), our data suggest that midbrain dopamine neurons can acquire predictive responses to negative reinforcers (e.g., warning signal predicts safety) and this learning mechanism might contribute to the development of conditioned avoidance. A longitudinal study assessing for changes in dopamine release to the warning and safety signals over training, would provide additional support for the role of temporal difference reinforcement learning in the acquisition of conditioned avoidance and offer clarification regarding the nature of the safety signal. As it stands, it is possible that the safety signal is more akin to a confirmation of shock avoidance/termination rather than a true signal of safety. Dopaminergic models of temporal difference reinforcement learning predict that dopamine neurons would stop encoding the safety signal as they begin to encode the warning signal. If the safety signal were a confirmatory signal, dopaminergic encoding of the safety signal should persist irrespective of training history. It is also important to note that we do not believe that such computational learning theories are at odds with psychological theories involving the role of dopamine in motivation. On the contrary, as previously described in detail (McClure et al., 2003) many commonalities between the reinforcement learning and motivation literatures exist.

Our conditioned avoidance model predicts that the warning signal is ultimately more important than the safety signal in promoting successful avoidance, as only the warning signal evoked-dopamine response predicts the behavioral outcome (i.e., avoidance vs. escape). It should be noted that this model is only intended to apply to operant signaled shock avoidance tasks. We still believe the mesolimbic dopamine system may function in Sidman operant avoidance tasks, where operant avoidance is maintained without an exteroceptive warning signal (Sidman, 1953), as an anticipatory timing signal (Bromberg-Martin et al., 2010)—although additional experiments are required to test this hypothesis. Also, certain factors of our theory (e.g., factor 2) might be more difficult to detect using a shuttle box because a directed instrumental response is not required for avoidance. Finally, we would like to add that the fourth factor of our model that the warning-signal evoked dopamine release actually promotes successful avoidance, is currently being experimentally assessed using optogenetic technology. These studies will directly test whether the role of dopamine in conditioned avoidance is causal or merely an epiphenomenon, and further discern if the role of dopamine in conditioned avoidance is related to reinforcement learning, motivational processes or, as we predict, both.

### Conflict of interest statement

The authors declare that the research was conducted in the absence of any commercial or financial relationships that could be construed as a potential conflict of interest.
